# Molecular Aspects of Regional Pain Syndrome

**DOI:** 10.1155/2020/7697214

**Published:** 2020-04-11

**Authors:** Manuela Baronio, Hajra Sadia, Stefano Paolacci, Domenico Prestamburgo, Danilo Miotti, Vittorio A. Guardamagna, Giuseppe Natalini, Stephanie G. B. Sullivan, Matteo Bertelli

**Affiliations:** ^1^Dipartimento di Anestesia, Rianimazione, Terapia Intensiva e del Dolore, Fondazione Poliambulanza, via Bissolati, 57, 25124 Brescia, Italy; ^2^National University of Sciences and Technology, NUST Campus, H-12, Islamabad, Pakistan; ^3^MAGI's Lab, Via delle Maioliche. 57/D, 38068 Rovereto, TN, Italy; ^4^Ortopedia e Traumatologia, Ospedali Civili di Legnano e Cuggiono, Via Papa Giovanni Paolo II, 20010 Legnano, MI, Italy; ^5^Cure Palliative e Terapia del Dolore, ICS Maugeri, Via S. Severino Boezio, 28, 27100 Pavia, Italy; ^6^Cure Palliative e Terapia del Dolore, IRCCS IEO, Via Adamello, 16, 20139 Milan, Italy; ^7^Dr. Sid E. Williams Center for Chiropractic Research, Office for Senior Health and Wellness, Life University, 1269, Barclay Cir, 30060 Marietta, GA, USA; ^8^MAGI Euregio, Via Maso della Pieve, 60/A, 39100 Bolzano, Italy; ^9^EBTNA-LAB, Via delle Maioliche, 57/B, 38068 Rovereto, TN, Italy

## Abstract

The purpose of this review is to summarize the pathophysiology of complex regional pain syndrome (CRPS), the underlying molecular mechanisms, and potential treatment options for its management. CRPS is a multifactorial pain condition. CRPS is characterized by prolonged or excessive pain and changes in skin color and temperature, and/or swelling in the affected area, and is generally caused by stimuli that lead to tissue damage. An inflammatory response involving various cytokines and autoantibodies is generated in response to acute trauma/stress. Chronic phase pathophysiology is more complex, involving the central and peripheral nervous systems. Various genetic factors involved in the chronicity of pain have been identified in CRPS patients. As with other diseases of complex pathology, CRPS is difficult to treat and no single treatment regimen is the same for two patients. Stimulation of the vagus nerve is a promising technique being tested for different gastrointestinal and inflammatory diseases. CRPS is more frequent in individuals of 61–70 years of age with a female to male ratio of 3 : 1. Menopause, migraine, osteoporosis, and asthma all represent risk factors for CRPS and in smokers the prognosis appears to be more severe. The pathophysiological mechanisms underlying CRPS involve both inflammatory and neurological pathways. Understanding the molecular basis of CRPS is important for its diagnosis, management, and treatment. For instance, vagal nerve stimulation might have the potential for treating CRPS through the cholinergic anti-inflammatory pathway.

## 1. Introduction

Complex regional pain syndrome (CRPS) is generally characterized by chronic pain, changes in skin color and temperature [1], edema in the affected area, localized sweating, and altered pattern of hair and nail growth [2]. Muscles become weak and the skin may appear glossy [2]. In later stages, the limb typically becomes cold. Tremor and dystonia may develop [3]. CRPS can be divided into two distinct subtypes. CRPS-I (previously known as reflex sympathetic dystrophy syndrome), without confirmed nerve injury, is caused by a fracture, bruise, or joint sprain combined with immobilization. CRPS-II (previously known as causalgia) is mainly causedbya peripheral nerve injury [4]. Since there is now evidence of neuronal injury in CRPS-I, as well [5], it is uncertain whether the two subtypes will be maintained.

CRPS is more frequent in individuals of 61–70 years of age with a female to male ratio of 3 : 1. Menopause, migraine, osteoporosis, and asthma all represent risk factors for CRPS and in smokers the prognosis appears to be more severe [6–8].

CRPS develops in response to injuries involving a limb (arm, leg, hand, or foot). It is a chronic pain condition that lasts more than six months. Changes in the central and peripheral nervous systems are involved in the pathogenesis of this syndrome [9, 10]. Symptoms may vary in degree, depending on the extent of tissue damage. In some cases, symptoms are mild and pass over a period of weeks, while in other cases they may be protracted. In severe cases, patients may not recover and may have a long-term disability [11].

This review aims to elucidate the pathophysiological basis and the role of genetics and the autonomic nervous system (ANS) in the development and management of CRPS. We also review the vagus nerve (VN) involvement in pain perception and how VN stimulation can help pain management.

## 2. Pathophysiology of Chronic Regional Pain

CRPS involves a set of maladaptive conditions characterized by continuing pain, either induced or spontaneous. The pain is regional; i.e., it does not involve a specific organ or nerve territory. Multiple pathophysiological mechanisms are involved in the development of CRPS, to which the central nervous system(CNS) is widely reported to contribute [10].

Although many attempts have been made to reduce CRPS to a single pathophysiological mechanism (e.g., sympatho-afferent coupling) [12], it is increasingly accepted that multiple mechanisms are involved. Apparently, CRPS is also a disease of the CNS. In fact, CRPS patients display changes in somatosensory processing of thermal, tactile, and noxious stimuli. Furthermore, the sympathetic nervous system changes are also observed in patients with unilateral CRPS symptoms and the somatomotor system may also be affected [10]. Based on the relative contribution of the pathological mechanisms involved, CRPS may exist in different subtypes [13]: CRPS-I, without a direct nerve injury, caused by a fracture, bruise, or joint sprain combined with immobilization; CRPS-II, caused by peripheral nerve injury [4]. Two main mechanisms have been proposed.Inflammation in response to trauma [14], in particular the neurogenic inflammatory component [15], and sympathetic nervous system dysfunction [16].

## 3. Inflammation in Pain and Role of Autoantibodies

CRPS patients show signs of inflammation, such as redness, edema, pain, and change in skin temperature, especially in the acute phase. This demonstrates that immune activation in posttraumatic patients can be a primary feature of CRPS pathophysiology [17]. The innate immune response triggers proliferation of keratinocytes and release of proinflammatory cytokines, such as tumor necrosis factor-*α* (TNF-*α*), interleukin 1*β*, and interleukin 6 [18]. These cytokines activate the connective tissue, leading to contractures [19]. They also act on osteoblasts and osteoclasts, leading to the high-turnover osteoporosis and bone loss associated with CRPS [20].

Cytokines also sensitize peripheral nociceptors and second-order neurons in the spinal cord, increase pain sensitivity (hyperalgesia), and facilitate the release of neuropeptides from primary nociceptive afferents [21]. Neuropeptides such as calcitonin gene-related peptide and substance P are released from cytokine-sensitized nociceptors and induce a phenomenon known as neurogenic inflammation, which could be responsible for the reddening, warmth, and edema in acute CRPS [22]. Another peptide, endothelin 1, contributes to cold, bluish skin [23]. In the course of CRPS, most of these signs normalize, which demonstrates a change in pathophysiology [3].

In addition to activation of the innate immune response and release of cytokines, there is also evidence suggesting a contribution of the adaptive immune response [24]. The role of autoimmunity and autoantibodies in response to injury has been studied by various researchers who have reported linkage of HLA class 1 antigens with the development of CRPS [25] and autoantibodies against autonomic nervous system structures [26]. Goebel et al. also demonstrated increased binding to various peripheral and CNS structures in CRPS serum [27]. However, the underlying antigens and pathophysiology are unclear. Kohr et al. found that 30–40% of CRPS patients have surface-binding autoantibodies against an inducible ANS autoantigen. These findings suggest that CRPS may involve autoimmune mechanisms [24].

Pathogenic autoantibodies targeting the ANS have been found in CRPS patients [24]. Tékus et al. confirmed the role of autoimmunity in an IgG-transfer-trauma mouse model of CRPS and found key clinical indicators of the human disease, namely, swelling and mechanical hyperalgesia, in the mouse model [28]. Further studies are needed to elucidate the origin and function of these autoantibodies in CRPS [24].

Further evidence in support of an autoimmune component involving dendritic cells comes from a study showing that IgG immune complexes stimulate migration of dendritic cells from peripheral tissue to draining lymph nodes *in vitro* [29]. The role of dendritic cells in autoimmunity has yet to be fully explored.

## 4. Role of the Autonomic Nervous System in Pain Regulation

Mechanical or thermal stress stimuli damage tissues, activating peripheral nociceptors. However, nociception does not always cause pain perception [30]. In CRPS patients, there is great variation in the warmth and sweating of the affected region, showing the involvement of local neurogenic inflammation [31], particularly in the acute phase, and disturbance of central thermoregulation [32]. The primary homeostasis of the body is mainly controlled by the ANS through balanced sympathetic and parasympathetic activity. Acute stress activates the sympathetic branch of the nervous system and causes an unbalance between sympathetic and parasympathetic activities [33]. In cases of chronic stress, the balance is disrupted for longer periods. In CRPS patients, there is an imbalance in ANS activity [34], and sympathetic dysfunction leads to clinical manifestations, including limb sweating, changes in skin temperature and color, and edema [3].

Activation of peripheral nociceptors activates nociception, the sensory nervous system's response to triggers. Nociception triggers the brain and spinal cord response, with or without pain perception; in fact, the feeling of pain does not depend on nociception [31]. Pain depends on interoception, the sense of the physiological condition of the entire body, not just the viscera [35].

Researchers have used noninvasive neuroimaging techniques to study nervous system activity in response to different nociceptors. They found that nociceptive stimuli generate responses in cortical and subcortical brain structures. Based on preferential involvement in pain perception and consistency across different studies, these brain structures have been clustered and named “pain matrix.” The pain matrix involved in pain generation includes the somatosensory (primary and secondary), cingulate, and insular cortices [36–39]. By measuring the activity in the cortical network (pain matrix), the actual feeling of pain generated by nociception can be established and quantified [40].

All the structures of the pain matrix are major constituents of the ANS and play an important role in emotional and cognitive processes. This shows the functional and anatomical link between the pain matrix and the ANS [41]. The ANS includes afferent and efferent nerve fibers. Thin afferent fibers are responsible for transmitting interoception from sensory receptors of internal organs, glands, and blood vessels to the spinal cord and other CNS structures. The internal environment is also monitored by central chemoreceptors, including those in the hypothalamus. Neuroanatomical analysis has revealed that the afferent fibers of the ANS are involved in conveying interoceptive information (e.g., touch and pain sensation) to the brain through the lamina I spinothalamocortical pathway involving cranial nerves, e.g., the vagus nerve [42]. Painful stimuli are conveyed somatotopically to both insulae of the brain [43]. This highly specialized organization of nociceptive information in these brain areas may serve a number of functions, particularly that of coupling pain with the most appropriate autonomic and affective/emotional states.

Sympathetic and parasympathetic nervous systems are the two main efferent components of the ANS and they relay information from the brain to every major system and organ in the human body. Interactions between different brain regions, including the medulla, pons, and hypothalamus, may support the generation of differential autonomic response to various physiological triggers and behavioral challenges [44]. Neurons modulate painful information at the spinal level directly on lamina I of the spinal cord [45]. Brain arousal and alertness, enhanced attention, and sensory processing of environmental stimuli promoted by the ANS play a crucial role in pain processing [46]. Alterations in the sympathetic nervous system contribute to CRPS. Understanding these alterations may be an important step towards providing appropriate treatments for CRPS [22].

## 5. Genetic Basis of Pain

Chronic pain, i.e., pain persisting for more than 12 weeks despite medication or treatment, affects approximately 30% of the population worldwide [47]. Genetic factors are a major risk for the development of chronic pain and contribute to the reported heritability of 16–50% [48, 49]. Researchers have delineated a centralized pain-processing system involving different neurotransmitters and the corresponding receptors. The system is modulated by growth factors and cytokines. Genetic studies have shown a strong association of pathological conditions with their alleged causal factors at the gene/protein level. Genetic factors are reported to play a role in CRPS, although familial occurrence has not been extensively studied. A study on the Dutch population reported that patients with familial CRPS developed the disease at a younger age with a severer phenotype than sporadic cases. Although no clear hereditary pattern was observed, the data suggests the existence of a genetic predisposition for developing CRPS [50]. Similarly, different association studies have revealed that individuals with different major histocompatibility complex (MHC) alleles are more susceptible to CRPS [51–53].

Numerous genetic risk factors have been identified for musculoskeletal, neuropathic, and visceral conditions. The genes involved in chronic pain include genes from different biochemical/cell pathways (catecholaminergic, serotonergic, estrogenic, glutamatergic, GABAergic, purinergic, and orexinergic), cytokines, growth factors, and proteinases. Rare but drastic mutations in single genes as well as polymorphisms in multiple genes have been identified as causative agents in different disorders and pain conditions.

### 5.1. Monogenic Disorders Associated with Pain

Variants in a single gene may cause pain disorders and can lead to aggravation of nociception or to a painless state. The latter class of disorders is linked to genetic loci involved in pain signaling or the viability of sensory neurons. These syndromes are helpful for understanding the pathophysiological mechanisms involved in pain processing. In fact, some of the same genes that harbor painful neuropathic variants also carry mutations leading to painless states [54]. For example, *de novo* mutations in genes encoding sodium channels, *SCN9A*, and *SCN11A*, have been reported in posttrauma pain perception and primary erythromelalgia [55, 56]. Erythromelalgia is characterized by redness and painful edema of the hands and feet, symptoms that have been attributed to C-fiber hypersensitivity [57]. Yang et al. found a link between primary erythromelalgia, the autosomal dominant hereditary form of the disease, and rare gain-of-function variations in sodium channel NaV1.7 encoded by *SCN9A* [58]. The study was later on replicated in Chinese and Caucasian patients by various groups [59–61]. The *SCN9A* variants implicated have been found to change the electrophysiological properties of dorsal root ganglion neurons, thereby affecting nociceptive signaling [62].

Variations in other genes have also been reported to be involved in peripheral neuropathies. For instance, the involvement of *α*-galactosidase A was reported in a small fiber neuropathy patient [63]. Similarly, the involvement of myelin protein zero was found in a family with debilitating neuropathic pain and demyelination. Patients with isolated peripheral nerve involvement with burning dysesthesias and pain at the distal segments of the 4 limbs have been reported to carry a mutation in a subunit of kinesin (KIF5A), a protein involved in intracellular motility [64]. A frameshift mutation in the TWIK-related spinal cord potassium channel (*TRESK*) gene that severely impairs the encoded protein's function has also been discovered in familial migraine conditions [65]. The effect of genetic contributors differs in neuropathic pain disorders in relation to the particular nerve dysfunction involved. In rare monogenic disorders, the somatosensory function is impaired by causal mutations in a single gene.

### 5.2. Rare Variants Associated with Chronic Pain

Chronic pain conditions are multifactorial disorders with a high frequency in the population. Common single nucleotide polymorphisms (SNPs) with a minor allele frequency of ≥1% in the general population are not directly the cause of chronic pain disorders but can modulate the susceptibility to them. The minor allele contributes to risk or protection by increasing (gain-of-function) or decreasing (loss-of-function) the activity of the resulting protein. While studying the association of genetic polymorphisms with pain perception, Reimann et al. genotyped 27 SNPs in the *SCN9A* gene. They observed a significant association between pain score and SNP rs6746030 in the general population and later an association with specific pain conditions. The rarer A allele, predicted to increase Nav1.7 (protein encoded by *SCN9A*) activity, was associated with increased pain scores with respect to the commoner G allele. Based on the particular genotype of *SCN9A*, individuals may experience varying degrees of pain in response to noxious stimuli [66].

Evidence of human leukocyte antigen involvement in the pathophysiology of CRPS has been reported by various researchers [67, 68]. A significant association of HLA-B62 and HLA-DQ8 alleles with CRPS patients with dystonia has also been reported, implicating these HLA loci in CRPS susceptibility and/or expression [69]. Dominguez et al. observed an increased risk of persistent postoperative pain after inguinal surgery in individuals carrying the HLA haplotype DRB1∗04–DQB1∗03:02. This suggests the involvement of homozygous/heterozygous DQB1∗03:02 alleles in neuropathic pain conditions [70].

Low back pain, another common clinical complaint, is associated with variations in the *CASP9* gene, a mediator of apoptosis [71, 72], and the matrix metalloproteinase 1 (*MMP1*) rs1799750 2G allele [73]. Additionally, polymorphisms in proinflammatory cytokine genes, mainly interleukin 1A [74, 75], interleukin 1 receptor antagonist [74], and interleukin 18 receptor subunits encoded by *IL18R1* and *IL18RAP*, are also associated with severity of lumbar disc degeneration, low back pain, and disability, as well as with the response to treatment [75].

Small fiber neuropathy has also been reported in patients with joint hypermobility syndrome (JHS)/Ehlers–Danlos syndrome (EDS) [76], delineating an association between small nerve fiber impairment and itch. An inherited connective tissue disorder, EDS has sometimes been reported in CRPS patients, suggesting that it had a role in the development of CRPS. The pathophysiology may include stretch injury of nerves passing through hypermobile joints, weakened connective tissue, or postsurgical nerve trauma [77].

By exome sequencing of a family affected with EDS, Martinelli-Boneschi et al. identified two rare variations in *COL6A5* cosegregating with a chronic itch in eight affected members and absent in nonaffected members. Two families and a diabetic patient carried the nonsense c.6814G > T (p.Glu2272^*∗*^) variant and another family carried the missense c.6486G > C (p.Arg2162Ser) variant. *In silico* analysis showed that both variants may have pathogenic effects. Results from in vitro studies revealed disorganization and reduction in COL6A5 synthesis, indicating an association between the *COL6A5* gene and chronic familial itch [78].

More studies are required to delineate the contributory genes in the regional pain syndrome.

## 6. Treatment Options

CRPS is a challenging chronic condition to treat successfully [79]. Due to its multifaceted pathophysiology, there is unlikely ever to be any general treatment for this syndrome. Because of the absence of effective medical treatments, palliative measures are often used, such as spinal cord stimulation. Pain relief options include physiotherapy, anti-inflammatory, and pain-relieving drugs (either oral, topical, or intrathecal administration). Other treatments may include directly blocking sympathetic nerves, surgical sympathectomy, and vagal nerve stimulation.

## 7. Pharmacological Treatments

For the treatment of CRPS, a number of drugs have been tested in order to find which one may be effective in pain relief. However, currently, only a class of molecules was proven to be effective in the management of CRPS: the bisphosphonates [80]. Bisphosphonates have potential pain killer activity by interfering with mechanisms involved in inflammatory and nociceptive pathways (inhibition of macrophage activation and proinflammatory molecules, regulation of nerve growth factor expression, and pH modulation in the site where the pain is localized) [81, 82]. The class of bisphosphonates comprises the following drugs: neridronate, which significantly reduces pain and improves the quality of life in patients with CRPS [83]; alendronate, which is effective for treating posttraumatic CRPS of the lower limb [84]; pamidronate, which may have positive effects on CRPS; and clodronate, which is effective in patients with CRPS-I at inducing pain relief [85]. All these bisphosphonates can be effective for CRPS in both research and clinical practice [83, 84, 86], without serious adverse events [87]. However, only neridronate provides clinically significant pain relief with complete and persistent remission in most cases of CRPS. Other classes of drugs that show contrasting results in relieving pain in CRPS patients are the following: opioids, calcitonin, anticonvulsants and antidepressants, nonsteroidal anti-inflammatory drugs, corticosteroids, anesthetics, antihypertensives, and botulinum toxin [88].

## 8. Neurological Treatments

### 8.1. Sympathetic Nerve Block

Sympathetic blockade involves the injection of local anesthetics along the lumbar sympathetic chain for lower limbs or stellate ganglion for upper limbs under fluoroscopic guidance. An increase in temperature in the affected extremity, without a motor or sensory block, a reduced pain, and a decreased allodynia are considered signs of a good response [89]. However, sympathetic blocks may be effective only in some patients, especially in those with mechanical allodynia with burning pain and with temperature and color changes [90–93]. Furthermore, there are no universally accepted guidelines for patients selection or drugs choice for regional blocks, and the quality of published reports on the lumbar sympathetic chain and stellate ganglion blocks is insufficient, with contrasting outcomes, some studies report benefits for the patients, and others show no effects and absence of consistency in the medications used [94–98].

### 8.2. Sympathectomy

In 1930, for the first time, a patient with CRPS was successfully treated through sympathectomy (stellate ganglionectomy) [99]. Sympathectomy may be chemical or surgical and may be successful in CRPS patients with sympathetically maintained pain, which already showed a good but transient effect from sympathetic blocks [89]. Since 1930, other clinicians have considered the sympathectomy as a feasible treatment for CRPS, particularly when undertaken in a timely manner (within 3 months). In this case, sympathectomy has an excellent or good outcome; otherwise, a favorable result is not guaranteed [100–103]. However, it was later proved to have variable outcomes, uncertain efficacy, frequent complications (neuralgia, hyperhidrosis, and Horner syndrome), and little evidence for long-term effects [89, 104–107]. For CRPS patients who show no improvement in long-standing symptoms despite medication, interventional or surgical therapy may be indicated [108].

Patients who respond to sympathetic nerve blocks are eligible for surgical or chemical sympathectomy. Sympathectomy can be either chemical, with the injection of phenol or alcohol performed with the same techniques and targets of sympathetic blocks, or surgical with an open approach or with minimally invasive techniques like thermoablation, which effects might be more lasting and reproducible [100]. Sympathectomy of the second thoracic ganglion may be undertaken with the thoracoscopic approach, a relatively easy and safe technique that leads to upper limb sympathetic denervation. The second thoracic ganglionectomy effectively interrupts the sympathetic outflow proximal to the alternate neural pathways [109–112]. Nerve regeneration frequently occurs after either surgical or chemical ablation but may take longer with surgical ablation [100]. Currently, guidelines about drugs selection are still missing; therefore, clinicians should carefully consider potential risks and benefits of these techniques before proceeding to the treatment [113]. High-quality evidence from double-blind randomized controlled trials with placebo controls would be needed to determine whether sympathectomy is actually effective in the reduction of neuropathic pain [108].

### 8.3. Spinal Cord Stimulation

The spinal cord stimulation (SCS) is based on the gate-control theory which proposes that “control of pain may be achieved by selectively activating the large, rapidly conducting fibers” [114]. This technique activates the nonnociceptive fibers, thereby preventing painful stimuli from reaching the thalamus, and is used in patients with CRPS who have already failed conservative management [115].

The SCS is an FDA-approved device made of electrode leads (percutaneous or paddle). Both percutaneous and paddle leads are placed at the midline in the epidural space to stimulate the dorsal column tracts of the spinal cord [116].

SCS controls the neuropathic pain of CRPS and modulates the pain mediated by the sympathetic nervous system. SCS may be effective for the treatment of CRPS in the decrease of pain and the increase in the quality of life [117, 118].

### 8.4. Vagal Nerve Stimulation

The vagal nerve is a major part of the autonomic nervous system that extends from the medulla to the colon and is involved in the autonomic, cardiovascular, respiratory, gastrointestinal, immune, and endocrine functions [119]. The VN is a mixed nerve containing both afferent and efferent fibers [120]. Its afferent fibers sense pressure, pain, stretch, temperature, chemicals, osmotic pressure, and inflammation [121] and are involved in regulating the homeostasis of the digestive tract and in the generation of heart and respiratory rhythms [122]. The efferent vagal fibers originate in the dorsal motor nucleus of the VN, located in the medulla. In humans, these fibers innervate the digestive tract, from the esophagus to the splenic flexure [123]. Through its afferent and efferent fibers, the VN plays a dual role in contrasting inflammation. Classically, vagal efferents are activated by peripheral proinflammatory cytokines (e.g., IL-1*β*) [124]. The anti-inflammatory reflex is mediated by the cholinergic anti-inflammatory response, in which the release of TNF-*α* is inhibited via the activation of alpha-7-nicotinic acetylcholine receptors on macrophages [125].

In case of pain, the stimulation of the VN can be effective in inducing antinociception. Noninvasive stimulation has been found effective in reducing acute pain [126]. Inhibition of somatic pain perception has also been observed with nonelectrical VN activation in clinical studies [127]. Since overactive sympathetic outflow has been associated with pain, pain may be reversed by reducing sympathetic outflow while increasing parasympathetic outflow. Together with the anti-inflammatory effect, autonomic modulation by vagal nerve stimulation can help manage pain [128] via suppression of pain neurons, as observed in animal models [129–131]. All these studies suggest a plausible role of vagal nerve stimulation in controlling and managing pain through multiple mechanisms. This nerve plays a pivotal role in the vagal-nociceptive network and vagal nerve stimulation seems promising for modulating nociception.

## 9. Conclusions

CRPS is frequent in individuals of 61–70 years of age with a female to male ratio of 3 : 1. Menopause, migraine, osteoporosis, and asthma all represent risk factors for CRPS and in smokers the prognosis is more severe. The pathophysiological mechanisms underlying regional pain syndrome are complex and involve both inflammatory and neurological pathways. Several approaches are being used for the treatment of this condition (pharmacological, sympathetic nerve blockade, sympathectomy, spinal cord stimulation, and vagal nerve stimulation) but further studies are needed. For example, the anti-inflammatory role of vagal nerve stimulation may be of particular interest and has shown great potential for treating pain. Furthermore, a better understanding of the molecular bases of CRPS will be important for the diagnosis, management, and treatment of this condition.
